# Adverse outcomes with extracorporeal adsorbent blood treatments in toxic systemic inflammation: a perspective on possible mechanisms

**DOI:** 10.1186/s13613-022-01078-6

**Published:** 2022-11-12

**Authors:** James Matson, Paul Lange, Patrick M. Honore, Kevin K. Chung

**Affiliations:** 1 Independent Consultant, Denver, CO USA; 2 Department of Medicine, Western Michigan University Homer Stryker School of Medicine, Kalamazoo, MI USA; 3 Medical Director, Donor Alliance, Inc., Denver, CO USA; 4 ICU Dept-Brugmann University Hospital, Faculty of Medicine of the ULB University, Brussels, Belgium; 5 Department of Medicine, Uniformed Services University, Bethesda, MD USA

**Keywords:** CytoSorb, Coupled plasma filtration and adsorption, CPFA, Adsorbent, Sepsis, COVID-19, Extracorporeal blood purification, Protein interface chemistry, Monoclonal antibodies, Bioreactor

## Abstract

**Background:**

Extracorporeal blood purification (EBP) treatments may be used in patients with sepsis and related conditions to mitigate toxic systemic inflammation, prevent or reverse vital organ injury, and improve outcome. These treatments lack demonstrable efficacy, but are generally considered safe. However, since late 2020, four clinical studies of EBP treatment using adsorbent devices in inflammatory disease reported significantly increased patient mortality associated with the adsorbent treatments. Criticisms of study design and execution were published, but revealed no decisive flaws. None of these critiques considered possible toxic effects of the adsorbent treatments per se.

**Perspective and conclusion:**

In adsorbent EBP treatment of systemic inflammatory disease the adsorbent media are deployed in patient blood or plasma flow for the purpose of broad spectrum, non-specific adsorptive removal of inflammatory mediators. Adsorption and sequestration of inflammatory mediators by adsorbent media is intended to reduce mediator concentrations in circulating blood and neutralize their activity. However, in the past two decades developments in both biomedical engineering and the science of cytokine molecular dynamics suggest that immobilization of inflammatory proteins on solid scaffolds or molecular carriers may stabilize protein structure and preserve or amplify protein function. It is unknown if these mechanisms are operative in EBP adsorbent treatments. If these mechanisms are operative, then the adsorbent medium could become reactive, promoting inflammatory activity which could result in negative outcomes. Considering the recent reports of harm with adsorbent treatments in diverse inflammatory conditions, caution urges investigation of these potentially harmful mechanisms in these devices. Candidate mechanisms for possible inquiry are discussed.

## Background

In critical illness resulting from toxic systemic inflammation, e.g., sepsis, COVID-19, extracorporeal blood purification (EBP) treatments have been deployed to reduce concentrations of inflammatory mediators (IM) in circulating blood. The goal of these treatments is to abate inflammatory organ injury and improve patient outcome. While evidence for efficacy of EBP treatment in acute inflammatory disease is lacking, it is generally considered safe [1].

However, since late 2020, contradictory findings have emerged: in four separate clinical studies, EBP treatment using either of two adsorbent devices was associated with significantly increased patient mortality risk. A hemoadsorbent bead device (CytoSorb^®^, CytoSorbents, Monmouth Junction, NJ) marketed in Europe was studied in three distinct patient groups: refractory respiratory failure due to COVID-19 requiring extracorporeal membrane oxygenation (ECMO) [2], circulatory shock following out-of-hospital cardiac arrest (OHCA) [3], and septic shock [4]. Combined plasma filtration and adsorption (CPFA, Bellco, Italy) using a resin adsorbent was studied in septic shock [5]. In each study, the adsorbent-treated group exhibited significantly increased mortality risk. Criticisms of the design and/or execution of the studies have been published, but no decisive clinical flaws were identified [6, 7]. No major toxicity for adsorbent EBP has previously been reported with either device in the clinical literature [8]. Therefore, potential mechanisms of harm will be sought outside the clinical domain, specifically in the domains of protein chemistry and bioengineering.

In this paper, we present the hypothesis that some blood or plasma inflammatory proteins may adsorb to a compatible solid matrix, e.g., beads, resins, membranes, functionalized fibers. For some proteins in some contexts, matrix adsorption may provide functional stabilization and presentation of reactive proteins in blood or plasma flows and thus promote their biologic function. Adsorption of these proteins to a large surface area matrix may amplify their function. These effects are contrary to the therapeutic intent of these EBP treatments. The presented hypothetical mechanisms of toxicity of protein adsorption-stabilization are intended to suggest initial avenues of laboratory and/or clinical investigation in order to promote safe and effective use of EBP treatments that involve adsorbent mechanisms.

### Recent evidence

Supady et al. performed a single-center, open-label, randomized, controlled trial in 34 patients with COVID-19 and severe respiratory failure requiring ECMO. Of these patients, 17 were also treated with hemoadsorption using the CytoSorb^®^ device, and 17 were controls (ECMO without hemoadsorption). Survival at 30 days was 3/17 (18%) in the hemoadsorption group, and 13/17 (76%) in the control group (*p* = 0.0016). Mortality hazard ratio (HR) with 95% confidence interval (95% CI) for adsorption treatment was 6.46 (1.64, 25.42), *p* = 0.0075 [2].

Akin et al. reported “early routine use of hemoadsorption” in 24 patients with circulatory shock following OHCA. Hemoadsorption patients were matched 1:2 to historic controls (*n* = 48); there was no difference in baseline parameters. In the hemoadsorption group, 30-day mortality was higher (83%) than in matched control subjects (65%, *p* = 0.011). Control patients were said to have “…a more favorable neurological outcome…” [3].

Garcia et al. prospectively recruited 48 septic patients with refractory shock (vasopressor dependency index ≥ 3 despite adequate volume resuscitation) and circulating IL-6 ≥ 1000 ng/L. Hemoadsorption treatment was started within 24 h of shock onset. These patients were matched to 48 historic control subjects. Intensive care unit (ICU) mortality was 42% in the control group, and 67% (*p* = 0.024) in the hemoadsorption group (mortality HR 1.82 [95% CI, 1.03–3.2; *p* = 0.038] [4].

Critics of these papers focused on study design, patient selection, and group balance [6]; and possible removal of beneficial solutes (e.g., antibiotics, immunosuppressors, antiepileptics, remdesivir) [7]. As important as these and other factors are, they do not include a key common element: the adsorbent devices themselves.

In these studies, adsorbent treatment was delivered using the CytoSorb device. The CytoSorb is a column packed with mesoporous beads through which blood is circulated resulting in broad spectrum, non-specific adsorption of IM molecules to the beads [9].

Hemoadsorption’s possible association with adverse outcomes is not unique. In coupled plasma filtration and adsorption (CPFA, Bellco, Italy) plasma is first separated from blood by a plasma filter, and the plasma is circulated through a styrenic resin adsorbent cartridge. Finally, the treated plasma is returned to the blood path downstream of the plasma filter; this combined plasma–blood flow undergoes hemofiltration and is then returned to the patient.

Garbero et al. reported a multi-center, randomized controlled trial of CPFA in adult patients with septic shock (COMPACT-2) [5]. CPFA target dose was 0.2 L/kg of plasma exchange over about 10 h each day. CPFA was started within 12 h of shock diagnosis and was discontinued after three consecutive days if shock had resolved. The first interim analysis revealed early deaths in the CPFA group; unplanned analysis revealed mortality was higher in the CPFA group (*n* = 63) at 3 days, than in the Control group (*n* = 52). Mortality at 3 days in the CPFA group was 30.2%, and in the Control group was 13.5%, *p* = 0.044; and at ICU discharge mortality in the CPFA group was 54%, and in the Control group was 28.8%, *p* = 0.008. In subjects without severe acute kidney injury mortality was directly related to the volume of treated plasma. A parallel trial of CPFA in septic shock (ROMPA) was terminated based on the COMPACT-2 findings [10]. Meta-analysis of both studies confirmed increased relative risk (RR) of early death with CPFA at day 3 [RR, 95% CI, 2.02 (1.14–3.59)], and day 28 [RR, 95% CI, 1.47 (1.03–2.10)] [11].

In the COMPACT-2 trial, two specific findings suggest resin adsorbent treatment may have contributed to the lethal outcome. First, the relative risk of death was highest by day 3 [RR, 95% CI, 2.24 (1.02–4.91); *p* = 0.044] and was the reason for trial termination. This close temporal association of early mortality with the start of CPFA treatment suggests a relationship between these events. Second, the dose–response relationship observed between mortality and the volume of adsorbent-treated plasma (*p* = 0.010), may implicate the resin adsorbent treatment.

The COMPACT-2 authors considered the combination of renal replacement therapy (RRT) with adsorption in CPFA could remove excessive antibiotics with lethal results in septic patients [5]. Indeed, the possible role of antibiotic removal in the excess mortality in all four studies must be considered.

For the sepsis studies [4, 5], anti-microbial drug removal is specifically relevant.

Reduction in the concentrations of anti-microbial drugs (meropenem, ciprofloxacin, piperacillin, flucloxacillin, gentamicin, vancomycin, voriconazole, fluconazole) in saline or albumin solutions by circulation through the CytoSorb device was measured in an in vitro recirculating test circuit [12]. Depending on the drug, concentration reductions ranged from 67 to 100% in the first 60 to 90 min of recirculation. In reconstituted human blood, by 30 min of recirculation, reduction of meropenem concentration was approximately 45% and reduction of ciprofloxacin concentration was approximately 52%. However, the test circuit fluid flow rate was 20 mL/min, which is much lower than the minimum CytoSorb blood flow rate of 100 mL/min in the clinical reports [2, 4]. A low fluid flow rate through the CytoSorb increases contact time between the drug and the adsorbent surface potentially enhancing adsorption and overestimating adsorbent capacity for anti-microbial drugs. [12].

In an in vivo model, CytoSorb’s impact on total body clearance of 17 anti-microbial drugs was “moderate” for fluconazole and linezolid, “mild” for liposomal amphotericin B, posaconazole and teicoplanin, and “negligible” for 12 of 17 other drugs [13].

In a retrospective clinical study in five septic shock patients treated with CPFA, piperacillin and vancomycin had high adsorbent extraction ratios (> 95%) across the resin early, but this decreased significantly by 8 h. Extraction of tazobactam was “low”. None of these drugs had significant changes in patient serum concentrations [14]. Thus, anti-microbial drug removal by CPFA is variable and may not affect serum drug levels.

Findings from in vivo studies for both CytoSorb and CPFA do not show consistent, clinically relevant anti-microbial drug effects.

In the study of hemoadsorption in patients with shock following OHCA [3], anti-microbial drug removal seems not relevant to the outcome.

For patients infected with COVID-19 remdesivir is a US Food and Drug Administration approved treatment. In an in vitro recirculating serum model, remdesivir and its active metabolite (GS-441524) were completely eliminated by CytoSorb treatment by 60 min [15]. However, remdesivir has no effect on mortality in COVID-19 patients requiring mechanical ventilation or ECMO [16] so its removal is unlikely to explain the excess mortality observed in such patients treated with CytoSorb.

Possible removal of certain anti-microbial drugs in critically ill infected patients by any form of EBP treatment requires close attention. The suggestion that adsorbent treatment may rapidly remove some drugs means therapeutic drug monitoring should be used and additional antibiotic doses should be considered, particularly early in treatment.

In the present reports [2–5] possible anti-microbial drug removal by adsorbent treatment appears to be either irrelevant [2, 3] or of doubtful significance [12–14], and so is unlikely to explain the reported excess mortality. Therefore, in an abundance of caution, inquiry into the adsorbent process per se as the source of toxicity is warranted.

### Conflicting evidence and possible resolution

As noted, adsorptive EBP is generally considered to be ineffective but safe [1]. Diab et al. [17] recently reported the first adequately powered, multi-center randomized controlled clinical trial investigating hemoadsorption efficacy in reducing severity of postoperative organ dysfunction in 288 patients undergoing surgery for infective endocarditis. Patients were randomly assigned to the hemoadsorption group (*n* = 142) or the control group (*n* = 146). Hemoadsorption was done using the CytoSorb device only during cardiopulmonary bypass; average hemoadsorption treatment duration was 2.31 ± 1.45 h. The primary outcome was the difference in the mean total postoperative sequential organ failure assessment score (ΔSOFA) and basal SOFA score. Secondary outcomes included 30-day mortality, postoperative stroke, duration of mechanical ventilation, vasopressor treatment, and renal replacement therapy, among other outcomes. There was no difference between groups in any outcome measure. Fifteen adverse events were monitored and were not different between groups.

In a smaller study, Stockmann et al. [18] reported a single-center randomized controlled clinical trial investigating hemoadsorption treatment with the CytoSorb device in adults (*n* = 49) positive for SARS-CoV2, with vasoplegic shock (the need for noradrenaline dose > 0.2 µg/kg/min to maintain mean arterial pressure ≥ 65 mm Hg), a C-reactive protein > 100 mg/L, and requiring continuous venovenous hemodialysis (CVVHD). The primary outcome was time to resolution of vasoplegic shock. Secondary outcomes were mortality at study day 7, day 30, ICU and hospital discharge; serum IL-6 on day 1 and 3 of intervention; duration of mechanical ventilation; duration of ICU-stay; and catecholamine dose on day 1, 2, 3, 7, and 30 after start of CytoSorb treatment. In the CytoSorb group (*n* = 23) treatment was initiated “right after” meeting inclusion criteria. The CytoSorb device was incorporated into CVVHD circuit before the dialysis filter and changed every 24 h; treatment continued for 3 to 7 days at the discretion of the treating physicians. An additional dose of antibiotics was administered with each CytoSorb device change. The Control group (*n* = 26) had no CytoSorb device in the CVVHD circuit. There were no significant differences between groups in any outcome measure or in occurrence of any adverse events.

In patients with toxic systemic inflammation, why should adverse events with protein adsorption treatment appear in some small studies with few treated patients, and not in others? Why should adverse events not appear in a large, randomized trial with many treated patients? The likely explanation is in the tenets of precision medicine that seek to identify patients who will, or will not respond to a treatment.

In response to infection or injury, the subject expresses its genetically determined humoral and cellular proteins supporting a distinct inflammatory response mechanism, or endotype. Endotypes determine, among other things, illness severity/organ dysfunction phenotypes [19], and response to therapy [20, 21]. If a septic patient’s endotype drives severe illness and includes potentially toxic proteins that are amenable to matrix adsorption and stabilization, and if the patient is treated with an adsorbent EBP, then inflammation and organ injury may be augmented and mortality increased. If the subject’s endotype does not include such proteins, then no inflammatory augmentation would occur with matrix adsorption.

A major disadvantage of small studies is their vulnerability to biased study groups. If the subjects in a small study were biased to endotypes amenable to enhanced toxicity with adsorbent EBP, then the toxic effect should be revealed. If bias to an endotype amenable to toxicity is lacking, then no enhanced toxicity with adsorbent EBP would appear. In larger studies where bias is minimized, mixed endotypes could minimize or mask toxic effects. In the terms of precision medicine, some patients may have endotypes favoring a response, albeit negative, to adsorbent EBP.

### Theoretical mechanisms of adsorbent injury in toxic systemic inflammation

Adsorbent devices in inflammatory disease are believed to sequester and neutralize IM thus effectively inactivating them. Does this reliably occur? Some of the most important chemical and physical processes in biology occur at a surface or interface between two phases, such as between circulating blood or plasma, and a solid, adsorbent surface. In this dynamic and complex domain, there could be effects that operate contrary to the therapeutic intent of the adsorbent treatment. Three of these theoretical mechanisms will be briefly discussed: stabilization and amplification of immobilized protein functions, depletion of critical protective species, and amplification of cytokine effects.

Stabilization and amplification of IM function is a relevant concern since IM are not removed from the blood circulation but remain on the adsorbent matrix and in the blood or plasma flow for the 10-to-24 h duration of therapy. Most adsorbed proteins are probably sequestered and inactivated, but some will be exposed on matrix surface to flowing blood or plasma.

**Fig. 1:**
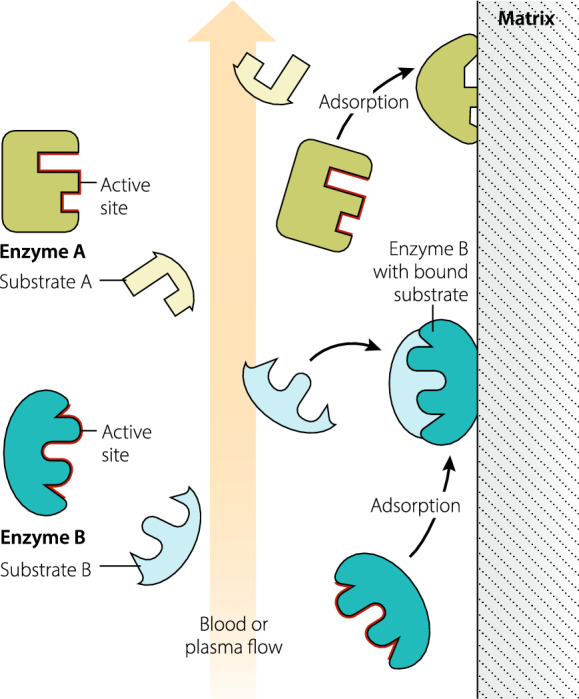
Stabilization and amplification of inflammatory mediator function. **Enzyme A** adsorption to matrix illustrates disrupted structure and a masked active site (AS) resulting in loss of function, i.e., the enzyme is totally neutralized. This is probably the fate of most enzymes adsorbed to matrix. **Enzyme B** adsorption to matrix illustrates stabilized structure and presentation of its AS in blood or plasma flow which could increase substrate binding events. Such enzyme function preservation or enhancement may be infrequent, but could be contrary to the therapeutic intention of adsorptive treatment

In biomedical engineering, adsorption of target proteins on solid or porous support matrices to create industrial bioreactors is established practice. In this application of interface protein chemistry, immobilization of the protein on a solid support stabilizes protein molecular structure and function, substantially prolongs its half-life, enables recovery and reuse of high value proteins, and can amplify function by enhanced protein presentation in process fluid flows (see Fig. 1). This expanding industry enables green and sustainable chemical synthesis in pharmaceuticals and other fine chemicals, as well as treatment of waste effluents [22, 23].

The most common proteins immobilized in catalytic bioreactors are enzymes. Several circulating enzyme systems are active mediators in toxic systemic inflammation. For example, acid sphingomyelinase (ASMA) is released into the circulation by lysosomal exocytosis from macrophages, endothelial cells, and other cells in response to pathogens, proinflammatory cytokines, or ligation of death receptors. In plasma, ASMA catalyzes sphingomyelin breakdown to ceramide, and ceramide amplifies the proinflammatory cytokine response. Ceramide also promotes membrane lipid raft formation which clusters receptor molecules; this amplifies signal transduction and apoptosis and promotes superoxide generation. High plasma ASMA activity correlates with disease severity and adverse outcome in COVID-19 [24]. In sepsis patients, ASMA plasma activity measured on the day of ICU admission and the day of discharge/death, significantly increased in nonsurvivors, and decreased in survivors (*p* < 0.02) [25]. Functional inhibitors of ASMA (FIASMA, i.e., marketed anti-depressant drugs) improve survival in murine sepsis models [26] and are used chronically in cystic fibrosis patients to control lung inflammation [27].

Granzymes (GNZ) are serine proteases with both cytotoxic and extracellular functions; extracellular functions are of interest here. In murine sepsis models, GNZ knockout improves survival. Plasma GNZ levels are elevated in human sepsis. GNZ, acting synergistically with endotoxin, potentiates cytokine (e.g., IL-1β, TNF-α, IL-6, IL-18) release from monocytes and fibroblasts, and processes cytokine precursors (e.g., pro-IL-1α, pro-IL-1β, pro-IL-18) into active molecules [28].

Adsorbent stabilization of ASMA in either blood or plasma, could enhance its activity on its substrate, sphingomyelin, generating ceramide and promoting proinflammatory activity. Stabilization of GNZ in blood, with exposure to endotoxin, monocytes, and fibroblasts, could promote proinflammatory activity. Both are contrary to the therapeutic intent of adsorbent treatment.

Removal of protective proteins, given the devices’ non-specific adsorption, is also a possibility. For example, proteases, given their destructive capabilities, require tight control by protease inhibitors. Inter-alpha inhibitor proteins (IαIp) are serine protease inhibitors of GNZ and other proteases, normally with high concentrations in human plasma. Lim et al. studied 51 patients with severe sepsis; controls were healthy volunteers. IαIp levels were inversely correlated with 28-day mortality rates and illness severity (APACHE II). In a murine model of *Escherichia coli* sepsis, intravenous administration of IαIp increased the 50% lethal dose by 100-fold [29].

Matrix metalloproteinase-9 (MMP-9), secreted by neutrophils, is involved in degradation of extracellular matrix, activates IL-8 in a positive neutrophil feedback loop [30] regulates cytokines and their receptors, and its plasma levels correlate with IL-6 [31]. Of interest and in contrast to ASMA and GNZ, in severe sepsis patients, *low* plasma levels of MMP-9 are associated with non-survival [32]. This may relate to the pivotal role of MMP-9 in the mobilization of circulating endothelial progenitor cells (cEPC) from the bone marrow [33]. Disruption of microvascular endothelium is a major mechanism of organ failure and death in severe sepsis. cEPC repair these defects; their circulating numbers correlate with survival in septic patients [34].

**Fig. 2:**
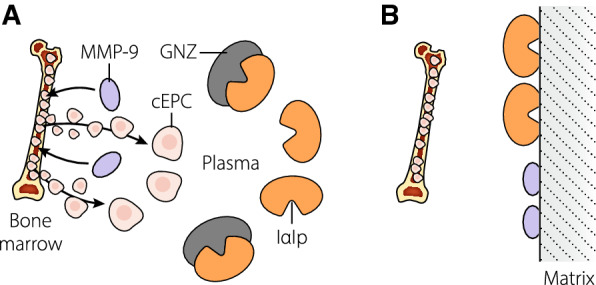
Removal of protective proteins. **A** Inter-alpha inhibitor proteins (IαIp) inhibit granzymes (GNZ) and other proteases reducing their toxic proteolytic activity. Matrix metalloproteinase-9 (MMP-9) transits to bone marrow where it mobilizes circulating endothelial progenitor cells (cEPC) to the circulation, promoting healing of microvascular endothelium. **B** Where matrix adsorption binds IαIp its activity may be reduced leading to disinhibition of proteases which may be injurious. Where adsorption binds MMP-9, mobilization of restorative cEPC may be blunted and their systemic restorative effects diminished or lost

Depletion of IαIp or other anti-proteases, or MMP-9 (see Fig. 2A, B), could inhibit damage control mechanisms in toxic inflammation, and so promote microvascular injury, organ failure, and death.

**Fig. 3:**
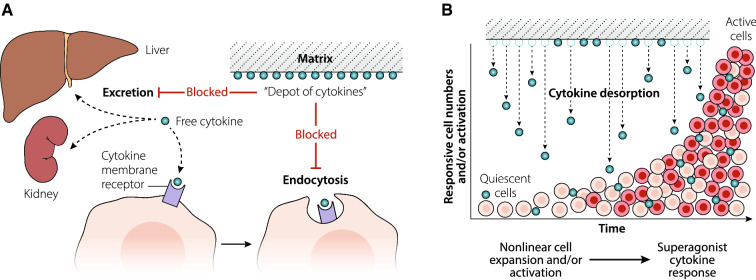
Cytokine rescue and depot. **A** The fast clearance of free cytokines principally results from renal and hepatic excretion and metabolism, and receptor clearance by endocytosis. Cytokine adsorption to a matrix could “rescue” the cytokine from renal or hepatic excretion and metabolism and prevent receptor binding and endocytosis. Cytokine adsorbed to matrix could form a “depot of cytokines”. **B** Gradual desorption of cytokine from the matrix “depot” over time could provide a sustained, low level of cytokine agonist to responsive cells. Nonlinear cell responses to sustained low cytokine levels could drive a “superagonistic” inflammatory response contrary to the therapeutic intention of adsorptive treatment

A third theoretical mechanism is amplification of cytokine effects. Plasma cytokine half-lives are typically very short, seconds to minutes; along with excretion and receptor consumption (see Fig. 3A), a short half-life is a needed restraint on these potent molecules.

Cytokine neutralizing monoclonal antibodies (mAb) have been developed as pharmaceutical control measures in several autoimmune diseases. Typically delivered in molar excess, several therapeutic mAb have successfully neutralized key cytokines effectively controlling disease activity in rheumatic and related disorders. However, for some cytokine–mAb combinations, at low mAb/cytokine molar ratios, binding of the mAb to its target cytokine creates “superagonistic” in vivo cytokine activity as much as 50- to 100-fold that of the free cytokine [35, 36]. Agonist activity enhancing mAb–cytokine complexes are reported for interleukin-2 (IL-2), IL-3, IL-4, IL-5, IL-6, IL-7, IL-15, tumor necrosis factor (TNF), and granulocyte-colony stimulating factor (G-CSF) [35–38].

Interaction of the mAb–cytokine complex with the cytokine receptor plays a minor role in activity amplification. The largest impact on amplification of cytokine agonist activity is attributed to the mAb acting as a carrier protein or “depot of cytokines”. Binding of the cytokine to the mAb results in a complex that ‘rescues’ the cytokine from fast clearance by excretion and receptor consumption; these effects stabilize the cytokine which materially prolongs its half-life. This cytokine depot gradually desorbs or releases cytokine over time providing sustained activation of target cells. Because the target cells exhibit non-linear response kinetics, slow desorption of cytokine from an adsorbent matrix could provide a sustained, low level of cytokine, that, in the proper context, can drive an often dramatically amplified agonist effect (see Fig. 3B) [35, 37, 38].

This cytokine depot mechanism may have played a role in the lethal effects in septic shock patients of the tumor necrosis factor receptor:Fc (TNFR:Fc) construct. TNFR:Fc had been safe and effective in rheumatoid arthritis patients, but in a randomized controlled trial in septic shock patients, TNFR:Fc exhibited a dose-related increase in 28-day mortality. TNFα was detectable in only 4% of patients at base line but was found in 40% of treated patients [39]. This suggests material stabilization and persistence in the patient’s circulation of TNFα by TNFR:Fc. Reported in 1996, this study may be an early example of the cytokine depot mechanism resulting in inadvertent amplification of inflammation with lethal effects.

Molecular binding characteristics of cytokines to mAb or to adsorbent matrix, no doubt differ in various ways. However, if the adsorbent matrix, in some contexts, effectively acts as a cytokine depot, then sustained release, desorption, of adsorbed cytokines may promote inflammatory organ injury and negative outcomes.

## Conclusions

Adsorptive EBP has been used in patients for more than 50 years to treat exogenous poisoning. These toxins are typically small molecules of < 500 Da. In this application, the paradigm of adsorption, sequestration, and effective toxin neutralization by the adsorbent medium has been successful and is generally accepted [40]. However, this neutralization paradigm may not apply to proteins which are much larger, and dynamically interactive.

Over the past 20 years, developments in protein interface chemistry and cytokine–mAb chemistry indicate that proteins are not necessarily neutralized by binding to adsorbent matrices or other proteins. Depending on context and the proteins involved, protein structure may be stabilized by an adsorbent matrix or carrier protein, and its function preserved or amplified. If these mechanisms became operative in EBP adsorbent media, then the adsorbent device could become an inadvertent bioreactor, promoting inflammatory injury, and negative outcomes. Similar dynamics may explain the lethal outcome of the TNFR:Fc trial.

Each of the mechanisms discussed in this paper is robust, effective, and being actively exploited by the bioengineering and pharmaceutical industries to deal with a variety of industrial and medical problems [22, 23, 41]. Thus, the mechanisms themselves, in their proper contexts, are well established.

The relevance of these mechanisms to adsorptive EBP is theoretical. However, the recent clinical reports of adverse outcomes with adsorptive EBP, with relative increases in mortality of 28 to 242%, means a reevaluation of adsorptive treatments is urgently needed. The mechanisms discussed herein are offered as plausible starting points for laboratory and clinical studies of adsorptive EBP; clearly, this list is not exhaustive. The goal of this reevaluation is enhanced safety and efficacy of adsorptive EBP.

## Data Availability

No datasets were generated or analyzed during the current study.

## Abbreviations

95% CI: 95% Confidence interval
APACHE II: Acute Physiology and Chronic Health Evaluation II
ASMA: Acid sphingomyelinase
cEPC: Circulating endothelial progenitor cells
COVID-19: Coronavirus disease 2019
CPFA: Coupled plasma filtration and adsorption
EBP: Extracorporeal blood purification
ECMO: Extracorporeal membrane oxygenation
FIASMA: Functional inhibitors of ASMA
G-CSF: Granulocyte-colony stimulating factor
GNZ: Granzymes
HR: Hazard ratio
ICU: Intensive care unit
IL: Interleukin
IM: Inflammatory mediators
IαIp: Inter-alpha inhibitor proteins
kg: Kilogram
L: Liter
mAb: Monoclonal antibody
MMP-9: Matrix metalloproteinase-9
OHCA: Out-of-hospital cardiac arrest
RR: Relative risk
RRT: Renal replacement therapy
TNFR:Fc: Tumor necrosis factor receptor:Fc construct
TNFα: Tumor necrosis factor alpha
